# Enhancing drug administration in *Drosophila melanogaster*: a method for using solid dispersions for improved solubility and bioavailability

**DOI:** 10.1080/19336934.2025.2497565

**Published:** 2025-04-25

**Authors:** Sunayn Cheku, Blase Rokusek, Mahesh Pattabiraman, Kimberly A. Carlson

**Affiliations:** a Department of Biology, University of Nebraska at Kearney, Kearney, NE, USA; b Department of Chemistry, University of Nebraska at Kearney, Kearney, NE, USA

**Keywords:** Drug delivery, *Drosophila melanogaster*, compound administration, solid dispersion, geldanamycin (GA), distearoylglycerol (DSG), RU486

## Abstract

*Drosophila melanogaster* is a widely used model organism for diseases such as Parkinson’s disease, Alzheimer’s disease, obesity, and diabetes. However, compound administration-based toxicological and behavioural studies on Drosophila have been hindered by technical difficulties associated with inefficient administration of hydrophobic compounds. This study illustrates a general method to make and distribute PEG 8000–based solid dispersions for three hydrophobic compounds, distearoylglycerol (DSG) geldanamycin (GA) and RU486 to *D. melanogaster*. The solid dispersions were validated, *in vitro*, using nuclear magnetic resonance spectroscopy (NMR), to have a higher aqueous solubility. The study also describes three different methods to administer the solid dispersions: subcutaneous injections, mixing in solid food, and the capillary feeder assay (CAFE). We show that the presence of 1% DMSO decreases survival, whereas PEG does not have an adverse effect. Lastly, we showed that the prepared PEG-RU486 formulation showed signs of enhanced bioavailability when compared to RU486 dissolved in ethanol. The methodology described in the study provides an easy and effective means to administer hydrophobic compounds to *D. melanogaster* using subcutaneous injections, CAFE assay, or by mixing it with solid food.

## Introduction

*Drosophila melanogaster* is the most widely used organism in animal model-based studies. They share approximately 75% of disease-causing genes with humans [1], are economically viable, have a short lifespan, produce a large number of offspring and are relatively easy to manage in a laboratory setting. Due to these advantages, they have been used as a model organism for diseases such as Parkinson’s disease, Alzheimer’s disease, obesity and diabetes [2–9]. The prevalence of various disease-model lines of *D. melanogaster* also makes it a suitable organism with which to conduct *in vivo* pharmacological and toxicological studies of novel drugs. Though they are suitable models for such studies, technical challenges related to mode of drug administration and suitable drug carriers limits their utility [10]. Therefore, there is a need for innovative approaches in terms of both drug administration and drug carriers to make pharmaceutical studies, as well as other studies involving compound administration, more feasible.

Drugs are broadly classified into 4 classes based on solubility and absorption rate: Class 1-high solubility-high absorption drugs, Class 2-high solubility-low absorption drugs, Class 3-low solubility-high absorption drugs, and Class 4-low solubility-low absorption drugs. There are challenges with the administration of each drug class in terms of formulating a carrier mechanism to maximize the drug bioavailability [11]. Approximately 70% of potentially new therapeutic drugs discovered in recent years tend to be hydrophobic and have low aqueous solubility, placing them in class 3 or 4. Maximizing bioavailability of such compounds requires methods that increase dissolution rate and absorption. Methods involving reduction of particle size, changing the crystal structure and state, using drug carriers and non-particle technologies are currently being used to solve this problem [12,13]. One such drug carrying method enhancing drug solubility and bioavailability is solid dispersion [14,15]. The term solid dispersion is used for formulations that include a hydrophobic compound with poor aqueous solubility, dispersed in a hydrophilic polymer matrix. Once the dispersion is introduced to an aqueous environment, the polymer dissolves, releasing the drug in the process. The hydrophobic drug that gets released shows an enhanced dissolution rate and absorption, thereby greater bioavailability [15,16]. While the exact mechanism of the enhanced dissolution rate is not completely understood, it is thought to be due to a combination of effects. First, a reduction in particle size causes an increase in surface area for absorption. Second, a change in the physical structure of the drug from crystalline to a more amorphous state promotes its dissolution [17]. A mathematical model to describe the mechanism of solid dispersions has been reviewed by Craig [18]. Though solid dispersions are effective, they have not been widely used to formulate commercial drugs due to long-term stability and bioavailability concerns. Additionally, the scale-up process for solid dispersion drugs is not as economically viable when compared to other methods [19]. This makes the method less favourable for large scale commercial applications. However, solid dispersions are a potential solution to the problem of administration of hydrophobic compounds to *D. melanogaster.*

The current methods to administer hydrophobic drugs to *D. melanogaster* are problematic. Widely used drug administration methods include vaporization, oral administration, and sub-cutaneous injections. While some drugs can be vaporized at a low temperature and pressure, the method is not practical for a wide range of drugs. Second, the sensitive chemoreceptors of *D. melanogaster* may hinder the oral consumption of chemicals having adverse tastes or smells [20]. Finally, injections require a suitable solvent formulation that dissolves the drug while having no toxic effects on the organism. Numerous factors, such as chemical interaction with the drug, viscosity, pH and metabolic effects, must be considered while choosing a suitable solvent. Though injections have been used to administer compounds to *D. melanogaster* in earlier studies, the compounds used were not highly hydrophobic, hence making them less problematic [21,22]. Dimethyl sulphoxide (DMSO) is a commonly used solvent to administer hydrophobic substances to organisms. However, previous studies show that it may have toxic effects at concentrations higher than 0.15–0.5% in *D. melanogaster* [23,24]. This would make its use problematic for studies evaluating behavioural or survival characteristics. Since solid dispersions are easy, and economically viable on the small scale, they could be an excellent potential method to administer hydrophobic drugs to *D. melanogaster* both orally and through injections.

**Figure 1.**
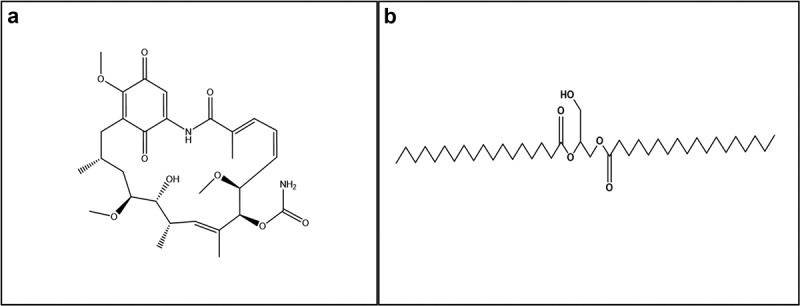
Molecular structure of (a) Geldanamycin (GA) (b) 1,2-distearoylglycerol (DSG).

This study evaluates the solubility characteristics of solid dispersions for two hydrophobic compounds, geldanamycin (GA) and 1.2- distearoylglycerol [18:0] (DSG) (Figure 1), dissolved in carrier molecule Polyethylene glycol 8000 (PEG) using nuclear magnetic resonance spectroscopy (400 MHz Bruker Avance NMR). The two compounds were chosen because of their highly hydrophobic properties. The two compounds being highly different structurally serves to illustrate the utility of PEG-based formulations in enhancing their aqueous solubility. We propose two potential modes of administration of the prepared solid dispersions through subcutaneous injections and oral feeding. Further, we have conducted a capillary feeder assay (CAFE) (Figure 7) and an agarose survival assay (Figure 8) to show that food consumption rate and survival is not negatively affected by the presence of PEG. To show the utility of solid dispersion formulations, a RU486-PEG solid dispersion formulation was tested. RU486 is a highly hydrophobic compound that is commonly used for activating Drosophila GeneSwitch systems [25]. A Drosophila line having a *GFP* gene regulated by a GeneSwitch system was used to compare the effectiveness of treatment with RU486 dissolved in 2% ethanol vs RU486-PEG formulation (Figure 9). We show that the enhanced solubility of the dispersion in an aqueous medium enables the preparation of homogenous mixtures for solid- or liquid-based oral drug delivery, as well as subcutaneous injection systems. Thus, this study demonstrates that solid dispersions could be an efficient, less toxic and cost-effective drug carrying mechanism to administer drugs to Drosophila and conduct various, pharmacological, toxicological and behavioural studies involving compound administration.

## Materials and methods

### Determination of drug to polymer ratio

It is well established that there is a general trend of increase in dissolution rates upon decrease in drug to polymer ratio in solid dispersion formulations. The increased dissolution rates are accompanied by increased drug bioavailability in studies where *in vivo* testing was conducted [26,27]. Furthermore, a drug: polymer (w/w) ratio of 1:9–1:3 was found to give the highest dissolution rates, while a high dissolution rate has been seen with a drug:polymer ratio of 1:20 for a PEG-based simvastatin formulation [28]. The highest dissolution rates were generally seen when drug to polymer ratio was lower than 1:3 for drugs, such as ibuprofen, azithromycin, chloramphenicol, sulfamethoxazole and ritonavir [27–31]. Hence, based on the existing literature, it is recommended to use a low drug:polymer ratio for optimal dissolution rates. Additionally, PEG 8000 was chosen as the polymer for this study, since it is a well-established non-toxic and biologically inactive polymer.

### Preparation of DSG-PEG solid dispersion using solvent evaporation method

DSG (Cayman chemical company, Ann Arbor, MI) and PEG 8000 (Sigma-Aldrich, St. Louis, MO) mixtures were prepared in different weight ratios (w/w) ranging from 1:6 to 1:15. Both the polymer and drug were weighed separately using a high precision weighing scale and combined to form a mixture. One millilitre of 99.9% chloroform was used per 1 mg of DSG to dissolve the mixture in a 2 mL glass vial. Chloroform was chosen as it readily dissolved both DSG and PEG in room temperature conditions yet retained a relatively low boiling point to facilitate evaporation. The solution was vortexed and sonicated until homogenous. A heating plate set at 40°C was used to dissolve the mixture if aggregates remained undissolved per the published method [31]. The solution was mixed for 6–12 hours at 30–45 RPM on a digital rocker to ensure incorporation of the drug into the polymer matrix. The solution was left to evaporate at room temperature. The solid dispersions were left to evaporate until the mass was measured to be constant using an analytical scale, to ensure minimal residual solvent. The leftover mixture was pulverized using a tissue grinder. The pulverized samples were used for drug administration and NMR testing.

### Preparation of GA-PEG solid dispersion

A geldanamycin (GA; Adooq bioscience, Irvine, CA) and PEG 8000 (Sigma-Aldrich) mixture were prepared in different (w/w) ratios ranging from 1:6 to 1:15 using a high precision weighing scale. Both the polymer and drug were weighed separately using a high precision weighing scale, and then combined to form a mixture. One millilitre of dichloromethane was used per 1 mg of GA to dissolve the mixture. Chloroform was not able to readily dissolve both GA and PEG 8000 at room temperature, however both the compounds dissolved in dichloromethane. The solution was vortexed and sonicated until homogenous. The solution was mixed for 6–12 hours at 30–45 RPM on a digital rocker to ensure incorporation of the drug into the polymer matrix. The solution was left to evaporate after mixing at room temperature. To ensure that minimal solvent residues remain, the solid dispersions were left to evaporate until the mass of the mixture was measured to be constant, using an analytical scale. The subsequent mixture was pulverized using a tissue grinder. The pulverized samples were used for drug administration and NMR testing.

### Preparation of RU486-PEG solid dispersion

RU486 (Sigma-Aldrich, St. Louis, MO)- PEG solid dispersions were prepared in a similar fashion to that of the DSG-PEG solid dispersion. A 1:8 (w/w) ratio of RU486-PEG was dissolved using chloroform as the solvent. The steps of preparation were similar to the one outlined above for the DSG-PEG dispersions.

### Microinjections

**Figure 2.**
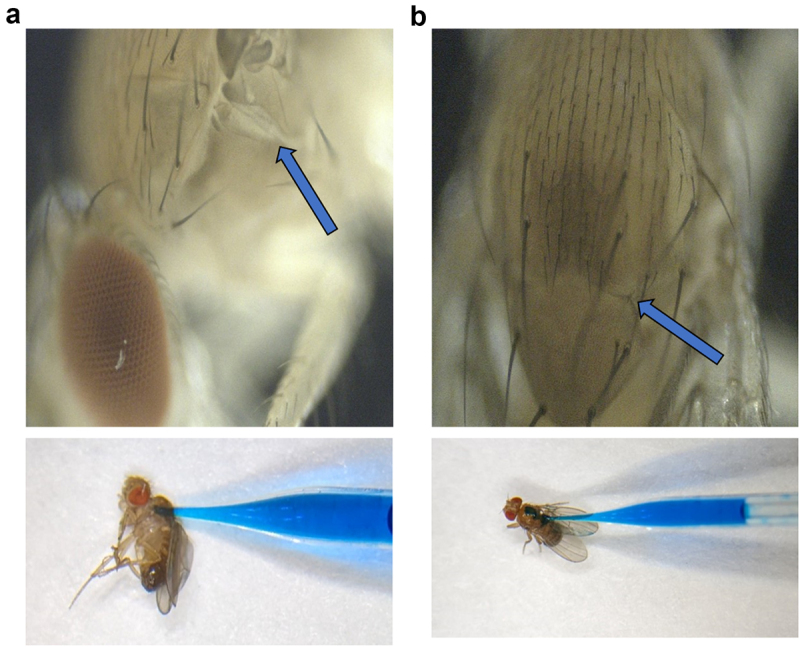
Site of injections on *D melanogaster*. (a) Left pteropleurite region. (b) Dorsal thoracic region.

The pulverized DSG-PEG dispersion was dissolved in 1 mL water with 5 µL of commercially available blue food dye to achieve a final drug concentration of 10 µM. The solution was loaded into custom glass needles prepared using a capillary puller (P30 vertical micropipette puller, Sutter instrument, Novato, CA) on 10 µL, 0.59 mm inner diameter glass capillaries. Groups of 5–10 *D. melanogaster* were anesthetized using ethyl ether for 15 s before being injected. The *D. melanogaster* was individually placed on a stereomicroscope and subcutaneous injections were performed using a FemtoJet 4i electronic microinjector (Eppendorf, Enfield, CT). A paint brush or metal forceps was used for stabilizing *D. melanogaster* during injections. The pteropleurite region (Figure 2(a)) and the dorsal thoracic region (Figure 2(b)) of *D. melanogaster* were found to be the most suitable spot for injecting in terms of ease and survival. Subsequently, the injections were done at pressures ranging from 100 to 300 hPa, depending on dosage requirements.

### Drug incorporation into solid food

The drug mixture obtained after filtration was dissolved in sterile deionized water to prepare a stock solution of 0.5–1 mm of the drug and 1 drop of commercially available blue food dye. The solution was mixed with instant fly food (Carolina Biological Supply Company, Burlington, NC), such that the final drug concentration in the food was 5 µM. The mixture was placed in vials containing 10–15 *D. melanogaster*. The Canton S wild type *D. melanogaste*r (Bloomington Drosophila Stock Center, Indiana University Bloomington, Cat#64349) used were maintained at 25°C under a 12:12 diurnal cycle.

### NMR

Proton Nuclear Magnetic Resonance (^1^H NMR) spectra were acquired on a Bruker Avance spectrometer operating at 400 MHz. The NMR samples were prepared in deuterated water (D₂O; Cambridge Isotope Laboratories, Tewksbury, MA). No internal standard was employed for referencing; instead, the solvent residual signal was referenced to 4.8 ppm (D_2_O/HOD). To ensure adequate signal-to-noise ratio even for low-abundance analytes, all experiments utilized a scan count of 5120. The recorded Free Induction Decay (FID) signals were processed using standard parameters within TopSpin software (version 3.1) for exponential apodization, Fast Fourier Transform (FFT), and phase correction. All NMR experiments were performed at an ambient room temperature ( ~ 21°C).

### CAFE assay

**Figure 3.**
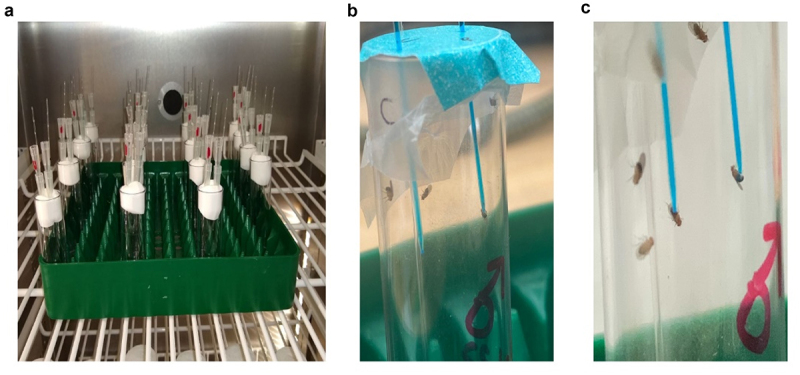
Capillary feeder (CAFE) assay. (a) Capillary tubes containing 5% sucrose arranged in the vial. (b) Magnified image of flies consuming drug incorporated 5% sucrose solution. (c) Vials placed in incubator set at 25°C under a 12:12 diurnal cycle.

The pulverized solid dispersion was added to a 5% sucrose solution to the desired drug concentration. The solution was loaded into a 10 µL glass capillary with an internal diameter of 0.59 mm. The capillaries were placed on top of vials holding 8 *D. melanogaster* each. Two different methods were utilized to insert the capillaries to the vials. The first design was built as previously described [32] (Figure 3(a)). The second design was built by placing a double layer of parafilm on the bottom and paper tape on the mouth of the vial. The paper tape strengthened the parafilm layer to support the capillary tubes. The loaded capillaries were placed through holes made on the double layer using a sharp glass capillary needle of the same diameter as mentioned above. An empty capillary was also inserted to allow air supply into the vials (Figure 3(b)). The latter design was found to be more convenient to build. The vials were placed in an incubator set to 25°C and 12:12 diurnal cycle to perform the assay (Figure 3(c)). The decrease in the liquid level in the capillaries was measured every 24 hrs to evaluate biases in food intake between experimental groups. Evaporation controls were used to account for decrease in liquid level. The evaporation levels were found to consistently be between 54% and 60% of the total drop in liquid level for all groups in experiments that were performed. The fall in liquid level due to evaporation was accounted for by standardizing the measures to their corresponding evaporation controls.

### Agarose starvation assay

A 1% agarose solution was prepared with deionized water. The prepared solution was adjusted to contain either 1% DMSO, 2% ethanol or 2% PEG. 10 mL of the respective solutions were added to plastic vials and left to solidify for an hour. Eight age matched pairs of male and female Canton S flies (3–4 days old) and placed in each of the vials. The vials were periodically checked every 6–8 hrs to count the number of flies that died during that interval.

### Treatment with RU486

A GFP-GeneSwitch line of *D. melanogaster* was used to test effectiveness of RU486 solid dispersions (Bloomington Drosophila Stock Center, Indiana University Bloomington, Cat#9431). Adult female flies were kept in vials containing instant fly food (Carolina Biological Supply Company, Burlington, NC) mixed with 1.2 mL water or 1.2 mL water containing 160 µM RU486 in a solid dispersion formulation or 160 µM RU486 dissolved in ethanol (final ethanol concentration in the food was 2%). The flies were cultured for 24 hours, after which their guts were dissected to perform confocal microscopy and check for GFP expression.

### Confocal microscopy

Guts of the GFP-GeneSwitch flies treated with RU486 were dissected out as described in detail in [33]. Care was taken to minimize exposure to direct light during all steps of the procedure to ensure retention of GFP fluorescence during imaging. The dissected samples were washed with 1X phosphate buffered saline (PBS) and fixed in 4% paraformaldehyde (PFA) for 20 mins. After fixation, the samples were washed with PBS three times for 5 mins each. The samples were permeabilized using 0.4% Triton-X dissolved in 1X PBS for 10 mins. The nuclei were stained using 1:1000 dilution of DAPI (Thermoscientific, Rockford, IL) in 1X PBS for 10 mins, followed by three 5 min 1X PBS washes. The samples were mounted on microscope slides using 20 µL of Vectashield mounting medium (Vector laboratories, Newark, CA). All imaging was performed using an Olympus FV3000 laser scanning confocal microscope (Olympus, Tokyo, Japan). Images were taken using a 20× objective. Images for each sample were taken using the same microscope settings using a 405 nm laser at 0.1% transmissivity and detection wavelength range of 430–470 nm for DAPI, and a 488 nm laser at 0.15% transmissivity and detection wavelength of 500–600 nm for GFP. All images were taken using a sequential scan mode to ensure minimal crosstalk between signals from the two fluorophores.

## Results

### NMR for evaluation of solubility characteristics of DSG-PEG dispersions

**Figure 4.**
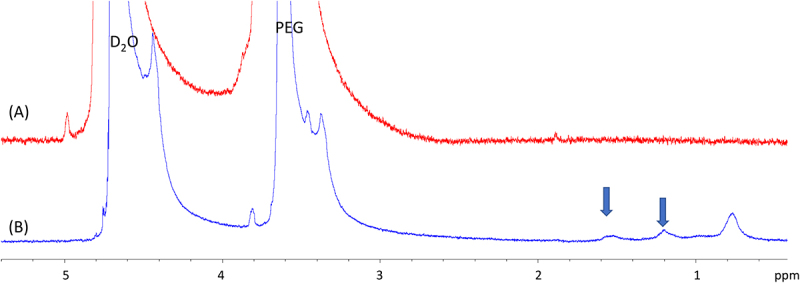
^1^H NMR spectra (a) 8 mg of PEG dissolved in D_2_O, and (b) that of filtered solution of 1 mg of DSG and 8 mg of PEG heated and sonicated for 2 hrs. DSG peaks are depicted with blue arrows.

NMR experiments were performed to observe the solubilization characteristics of DSG in aqueous medium achieved using PEG and extrapolate the understanding for studying its solid-state dispersion. DSG is a highly non-polar molecule and due to this, its solubility in an aqueous medium is low. After 2 hours, saturated aqueous solutions prepared by adding 1 mg of DSG to 1 mL of water/D_2_O, were sonicated and heated. To solubilize the compound in aqueous medium, we utilized PEG 8000. To a vial holding 1 mL D_2_O and 8 mg of PEG,1 mg of DSG were added and subjected to sonication for 2 hrs with heating. The resulting solution showed signs of dispersion of DSG as evidenced by visible cloudiness and distribution of particulate matter suspended evenly in the medium. The solution was filtered through a 0.2 mm filter tip with a syringe to obtain a filtrate free of insoluble particulate matter. The resulting filtrate was analysed on a 400 MHz Bruker NMR on a ^1^H pulse program with 16 scans, which showed broad, featureless signals in the up-field region of the spectrum that is attributable to the alkyl protons in DSG between 0.5 and 1.8 ppm; strong signals corresponding to D_2_O at 4.8 ppm, and PEG at 3.6 ppm, were also observed (Figure 4). The peak broadening observed for DSG is consistent with the effect observed during a chemical encapsulation, wherein the observed NMR signal of DSG is a weighted average of signals from a dynamic DSG in a directionally heterogenous PEG matrix. To confirm that the newly present signals corresponded to DSG, we performed bi-phasic extraction of Figure 4 solution B, wherein the particle free filtrate was equilibrated with CDCl_3_ for 5 mins with agitation produced from a vortex mixer.

**Figure 5.**
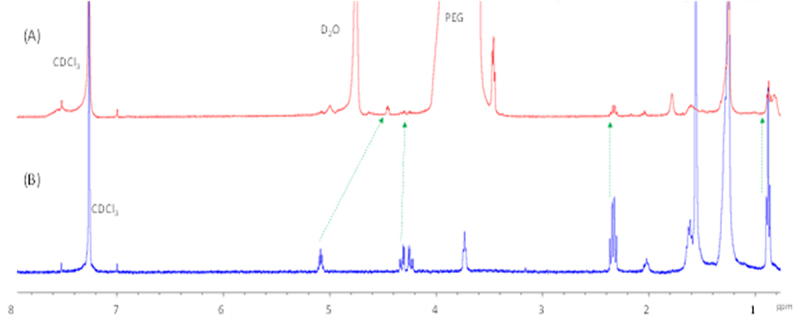
^1^H NMR spectrum of DSG (a) CDCl_3_ layer from the biphasic extraction performed with a saturated solution of DSG solubilized using PEG in D_2_O. (b) ^1^H NMR spectrum of DSG in CDCl_3_. Green arrows indicate DSG peaks.

Analysis of the CDCl_3_ layer extracted from our experiment showed well-featured DSG signals that correlated with the signals of an independently prepared solution of the compound in CDCl_3_ with slight up-field shifts in some of the signals, presumably due to perturbations caused by D_2_O and PEG (Figure 5). This indicated that PEG effectively solubilized DSG in an aqueous medium, which is extractable with CDCl_3_ at least partially. As a negative control, we performed the same experiment as described above without the presence of PEG (data not shown). The solution was filtered using a filter tip to remove insoluble particulate matter and subjected to the CDCl_3_ biphasic extraction. NMR of the CDCl_3_ layer did not show presence of any signals other than that of D_2_O for the same number of scans, which confirmed that the solubilizing agent, such as PEG, is necessary for its solubility.

### NMR for evaluation of solubility characteristics of GA-PEG dispersions

**Figure 6.**
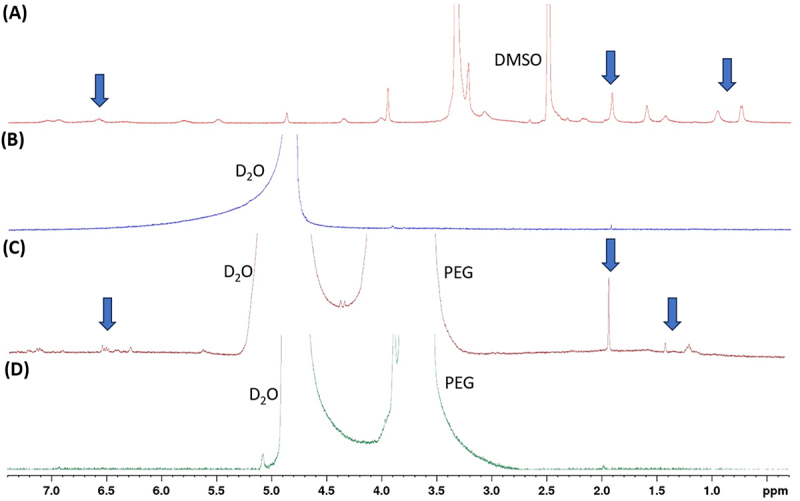
^1^H NMR spectrum of GA (a) 1 mg of GA dissolved in DMSO. (b) 1 mg of GA dissolved in D_2_O. (c) 1 mg of GA in 6.5 mg PEG solid dispersion in D_2_O. (d) 6.5 mg PEG dissolved in D_2_O. GA peaks are depicted with blue arrows.

The GA solubility profile in water and the effect of PEG solubilizer is shown in Figure 6. The full NMR spectrum of GA was completely soluble in DMSO and was recorded to visualize the proton signals of the compound for reference. This showed the presence of several signals between 0.5 and 7.5 ppm corresponding to the complex structure of the molecule and presence of a wide range of functional groups in the molecule ranging from alkoxy, to ketone, to amide groups (Figure 6(a)). The NMR (5028 scans) of an aqueous solution of GA added to D_2_O, sonicated, heated, and filtered, did not show any signals despite the high scan count indicating that GA was practically insoluble in water (Figure 6(b)). However, the NMR of a dispersion of GA with 6.5 mg of PEG, showed emergence of several new signals (Figure 6(c)) roughly corresponding to the signals seen in DMSO NMR spectrum of GA. These new signals in aqueous media were slightly shifted and broader compared to the DMSO signals presumably due to the change in medium and interaction of the PEG-dissolved GA, resulting from a heterogeneous solvation environment. This heterogeneity in the solvation environment likely leads to varying degrees of shielding and deshielding of the GA protons, causing the observed signal broadening in the NMR spectrum (Figure 6(c)). The spectrum of PEG alone dissolved in D_2_O showed presence of only PEG and solvent, confirming the newly emerged signals are due to the aqueous borne GA solubilized by PEG (Figure 6(d)). Additionally, in order to test the potential long-term stability of the GA-PEG formulation, an accelerated stability test was performed where the sample was heated at 55°C for 1 hour. The subsequent NMR performed showed similar peaks as the original dispersion (Supplementary Figure S1).

### Qualitative assessment of solid dispersion administration through solid food and injections

Blue food colour was added to the homogenous drug solutions prepared, served as indicators to ensure that the drug was being consumed and getting into the system of the flies. Light blue stains seen localized to the abdominal region of the fly served as a qualitative marker for both oral (through solid food) and subcutaneous form of drug administration. A direct quantitative assessment of the amount of drug mixed solid food, entering the system of individual flies was not able to be conducted due to technical challenges. Hence, a CAFE assay was conducted to address this issue. Additionally, 100% survival was observed upon treatment with 5 µM GA for both solid food and injections over a period of 96 hours (data not shown). It must be noted that flies that did not wake up post injections due to injuries were discarded, and therefore not a part of the survival assays conducted.

### Food intake analysis using CAFE assay

**Figure 7.**
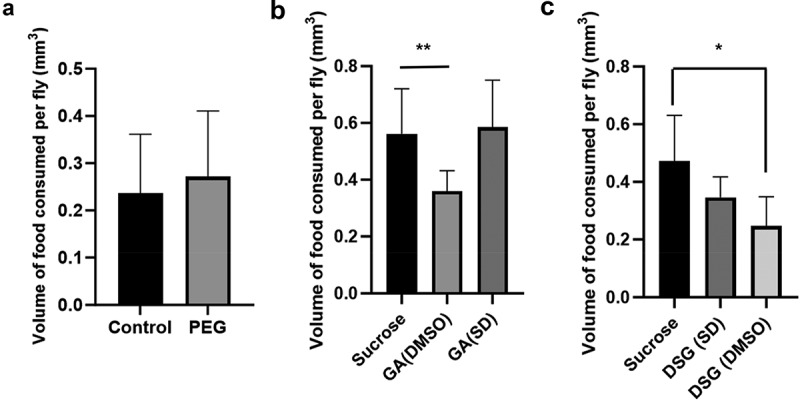
Food intake was assayed by using 5% sucrose solution and 5% sucrose solution with PEG. Results are expressed as an average ( ± SD) amount of solution consumed per fly in 24 hrs from 32 vials, each containing 8 flies (4 female and 4 male) of similar age. Statistical significance was determined by Welch’s *t*-test between 5% sucrose and 5% sucrose + PEG groups (*p* = 0.2887), which showed that there was no significant difference between the two groups. (b) 4 vials of 8 female flies of similar age were given either 5% sucrose, 5% sucrose containing 5 µM GA dissolved in DMSO, or 5% sucrose containing 5 µM GA in solid dispersion (SD) form. (c) 4 vials of 8 female flies of similar age were given either 5% sucrose, 5% sucrose containing 20 µM DSG dissolved in DMSO, or 5% sucrose containing 20 µM DSG in SD form statistical testing performed using a Welch’s *t*-test found a significant difference between both GA, and DSG dissolved in DMSO when compared to their no treatment counter parts (*p* = 0.0033 and *p* = 0.0358, respectively).

CAFE experiments showed no evidence for negative effects on food consumption due to the presence of PEG. The volume of food consumed in 24 hrs between the 5% sucrose solution group, and the 5% sucrose solution with PEG added to it were not statistically different, when compared using Welch’s *t*-test (*p* = 0.2887; Figure 7(a)). Additionally, there was no observed difference in the survival rates between the two groups, with both groups showing nearly 100% survival rates in all 32 vials over a period of 96 hours (data not shown). The DMSO groups were found to have a significantly lower consumption for both GA (Figure 7(b)) and DSG (Figure 7(c)) when compared to the no treatment sucrose groups analysed using Welch’s *t*-test (*p* = 0.0033 and *p* = 0.0358, respectively). All three groups also showed a 100% survival rate over a period of 96 hours (data not shown).

### Agarose starvation assay

**Figure 8.**
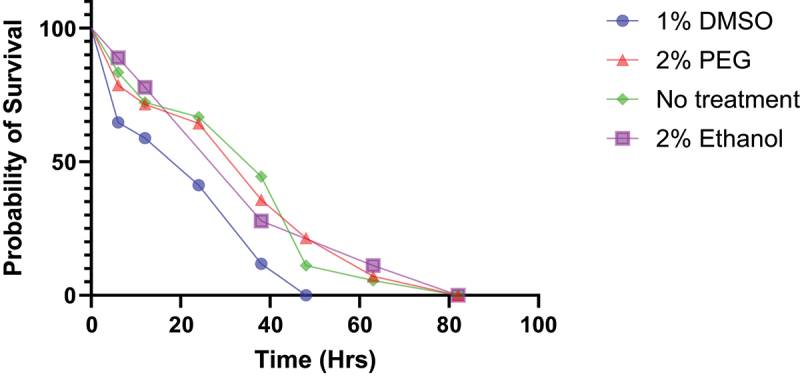
Survival curve of agarose starvation assay. Age matched pooled males and females (3–4 years old) were kept on vials containing 1% agarose only or 1% agarose with 1% DMSO, 2% PEG, or 2% ethanol (*n* = 28–32 flies per group). For clarity standard deviations have not been shown. Statistical significance was determined using a Log rank test.

By using a log rank (Mantel Cox) test performed between individual treatment groups, the agarose starvation assay showed a significant decrease (*p* = 0.0106) in survival in flies that were treated with 1% DMSO (Figure 8). The median survival period for flies treated with DMSO was found to be approximately 31 hrs compared to 38 hrs for 2% PEG and 2% ethanol and 48 hrs for untreated flies. While there was a noticeable difference in median survival times with PEG and ethanol treatments when compared to no treatment, the differences were not statistically significant (*p* = 0.127 and 0.23, respectively).

### GFP expression post RU486 administration

**Figure 9.**
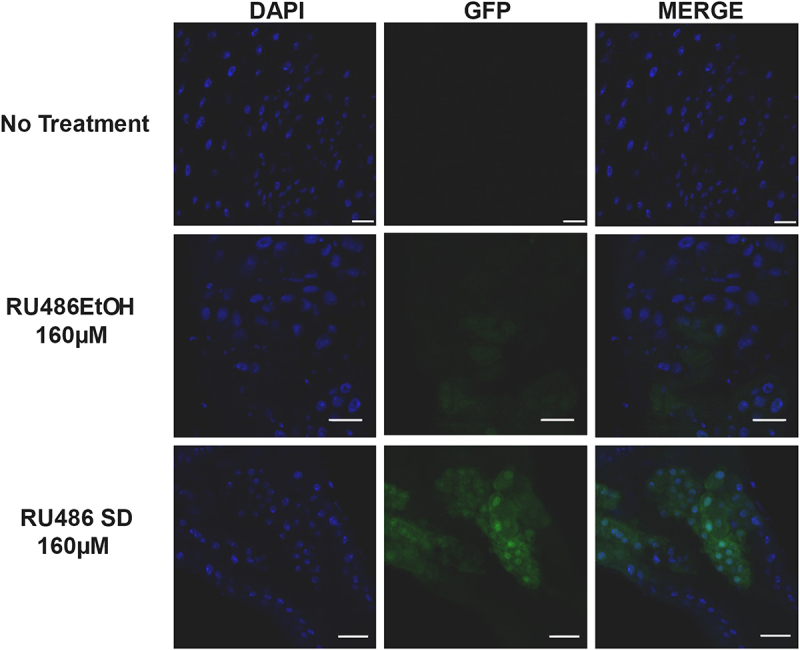
All images were taken using a 20× objective. (a) Fly gut section with no RU486 treatment, (b) Fly gut section treated with 160 µM RU486 dissolved in EtOH. (c) Fly gut section treated with 160 µM RU486 in solid dispersion formulation. DAPI staining for nuclei (blue), GFP (green).White bar represents 20 µm scale.

Confocal imaging was performed on gut tissue samples of flies expressing GFP on exposure to RU486. Flies were treated with 160 µM RU486 either using a solid dispersion formulation or dissolved in ethanol and diluted in water for 24 hrs. The flies that were treated with the solid dispersion formulation showed more intense signals for GFP as compared to those treated with RU486 dissolved in ethanol (Figure 9(b,c); Supplementary Figure S2). As expected, the gut samples of flies not treated with RU486 showed minimal signals for GFP throughout the gut tissue (Figure 9(a)), even with increased laser transmissivity (data not shown). These results were consistent through multiple samples with a representative sample shown (Figure 9). To verify the absence of the GFP signal, we increased the laser transmissivity setting to twice the original, and this served as an important check to ensure that the fly line used had non-leaky GFP expression in the gut tissue.

## Discussion

To validate the utility of solid dispersions in carrying hydrophobic drugs in *D. melanogaster*, two compounds were used, GA and DSG. GA is a hydrophobic drug with low absorption levels (Class 4) classified as a heat shock protein 90 (Hsp90) inhibitor [34], and DSG is a lipid that is highly hydrophobic. The significantly different chemical structures and properties of the two compounds serves to illustrate the widescale utility of PEG-based solid dispersion systems in potentially increasing the bioavailability of different hydrophobic compounds. NMR experiments were independently conducted to deduce the solubility profile of DSG and GA in their neat solid states, and enhancement of solubility affected by presence of PEG in aqueous media. NMR is an effective spectroscopic method to observe the aqueous solubility of substances as proton signals of analyte is observable only when it is completely soluble in the medium. The presence of proton signals for a given analyte indicates its solubilization in the medium, and the intensity of signal (area under the curve) indicates proportional concentration. Despite sufficient sonication and high scan setting, no noticeable DSG peaks were observed in the NMR spectrum, which indicated that DSG is practically insoluble in water. However, when 8 mg of PEG was added, the NMR of the resulting solution showed presence of PEG signals (Figure 4(b)) alongside the DSG signals for the same number of scans in the highfield region (0.6 to 1.6 ppm) of the NMR spectrum corresponding to the alkyl protons in the molecule. As a control, the NMR of PEG alone was also recorded for the same number of scans, and no peaks other than the PEG signals were present (Figure 4(a)), confirming our inference that PEG was effective in solubilizing a highly non-polar molecule such as DSG. The enhanced solubility of DSG using solid dispersion supports the utility of solid dispersions for carrying highly hydrophobic drugs. To confirm that the newly present signals corresponded to DSG, we performed bi-phasic extraction wherein the filtered D_2_O solution was equilibrated with CDCl_3._ The resulting H^1^ NMR signals were found to be correlated with the signals of DSG dissolved in CDCl_3_ (Figure 5). Similarly correlated signals were also seen between GA dissolved in DMSO and that dissolved in D_2_O while using PEG (Figure 6). Showing that the presence of PEG enhanced the solubility of GA in D_2_O.

Though this study used the solvent evaporation method for preparing the solid dispersions, there are other methods to prepare them, such as fusion or melting, and spray drying. The various methods have been reviewed in depth [16–18]. The choice for the method of preparation would largely depend on the chemical properties of drug and its interaction with the polymer structure. For instance, while using fusion method, problems may arise with miscibility of carrier and drug. Additionally, the fusion method is not favourable with drugs likely to undergo degradation at fusion temperatures. Similarly, large amounts of solvent used to dissolve the drug-carrier mixture in the solvent evaporation method may lead to toxicity issues due to residual traces of solvent if the solvent is not highly volatile. Consequently, the organic solvent utilized for the solvent evaporation method should readily dissolve both the drug and the polymer, be volatile, have minimal toxic effects, and undesired interactions with the drug-carrier mixture [16,17,35,36]. Both chloroform and dichloromethane seem to be suitable solvents from our testing as we saw no signs of increased toxicity in the SD formulations. Additionally, a suitable polymer must be selected. This would again depend largely on the chemical properties of the drug itself. Aside from PEG, several other polymers such as polyvinylpyrrolidone (PVP), cyclodextrins, and sorbitol have been used in previous studies [27–29,37,38]. PEG was selected due to its non-toxic, biodegradable and non-aversive taste and smell towards *D. melanogaster* [39]. Similar to these studies, no signs of toxicity or aversion to PEG were observed in the *D. melanogaster* at the concentrations that are required for solid dispersions (Figure 7).

As mentioned previously, *D. melanogaster* chemoreceptors are incredibly sensitive, giving them the ability to detect minute quantities of compounds and avoid them. Inconsistent uptake of drug-containing food may have confounding effects during experiments [40]. Therefore, it is important to ensure that the presence of the drugs incorporated into the food is not interfering with overall food consumption. To our knowledge the CAFE assay to monitor liquid food intake, and the consumption-excretion (CON-EX) assay to monitor solid food intake, are the most reliable food consumption assays [32,40]. The CAFE assays showed that neither the presence of PEG nor 5 µM GA in the solid dispersion form has an appreciable impact on the amount of food eaten by *D. melanogaster* (Figure 7). Additionally, a previous report suggested that RU486 decreases overall food consumption in Drosophila [41]. While we did not conduct a CAFE assay for the RU486-SD samples, the decreased aversions to SD samples as compared to their DMSO counterparts for DSG and GA suggests that RU486-SD samples could potentially mitigate some of the food consumption issues of RU486. This could potentially have some positive implications for the widely used Drosophila GeneSwitch systems.

A quantitative analysis of the amount of drug being delivered into the flies was not done for oral administration of the solid dispersions mixed with solid food due to technical challenges associated with it. However, we were able to qualitatively determine that the drug mixed solid food was entering the system by visualizing the blue food colouring localized to the abdominal region. The same was also done for drug administration through the injection route. Lastly, based on extensive trials, we found that the dorsal thoracic and the pteropleurite region of the fly were the best sites for sub-cutaneous injections based on both ease of injections and survival post treatment (Figure 3).

Furthermore, we saw no evidence of decrease in survival in flies that were treated with PEG both orally, and subcutaneously. This is again supported by results of previous studies that used PEG coated nanoparticles on *D. melanogaster* and saw no negative impacts on survival due to its presence [42–44]. A 2024 study, which evaluated the use of different polymers for nano particle preparation, found that PEG does not have a significant negative effect on long-term survival unless the total PEG concentration in the food is greater than 1% [45]. This finding is further supported by our agarose starvation assay (Figure 8), which found no significant difference in survival between 2% PEG treated and untreated flies. Since solid dispersion formulations do not require a total PEG concentration of greater than 0.1% for most Drosophila-related experiments, it is unlikely to have a significant negative effect on their long-term survival.

Additionally, our study supports the argument that the conventional method of dissolving compounds to DMSO is more likely to have negative consequences on fly lifespan. Our finding that 2% ethanol does not seem to induce a significant difference in survival from the agarose starvation assay (Figure 8), agrees with previous findings that ethanol is not toxic up to concentrations of 4% in fly food [46]. The nutritive value provided by ethanol could also provide an explanation for the absence of significant decrease in survival. However, the lack of toxicity does not necessarily correlate with significant enhancement of bioavailability. To test this, we compared treatment of 160 µM RU486 dissolved in ethanol with a 160 µM RU486-PEG formulation on a GFP expressing Drosophila GeneSwitch line. The subsequent confocal images taken of dissected gut tissue demonstrated a qualitatively enhanced presence of GFP signals from the RU486-PEG formulations possibly indicating an increase in bioavailability of RU486 (Figure 9). Additionally, increase in food palatability of RU486-SD vs RU486-DMSO could also be a contributing factor in the enhanced GFP signals we saw. Nevertheless, these results could have important implications for enhancing experimental results of studies involving the widely used RU486 activated Drosophila GeneSwitch systems.

Though a long-term stability test of the prepared solid dispersions were beyond the scope of this study, we performed an accelerated heating test of the GA-PEG formulation, which showed no change in the GA peaks present in a freshly prepared sample. While long-term stability of solid dispersions has been a concern for commercial pharmaceutical applications, studies have shown that PEG-based formulations remain stable for at least a period of 3–6 months, and potentially longer under appropriate storage conditions [31,47–49]. It must also be noted that high humidity levels are especially a concern for PEG-based formulations; hence, storage in low humidity environments and airtight containers is advised for long-term storage [50]. However, the above findings are unlikely to be a barrier for most Drosophila-related experiments, provided that experiments are performed within a reasonable timeframe post the preparation of the dispersions.

The administration of hydrophobic compounds in *D. melanogaster* is technically challenging. Development of novel methods to effectively administer hydrophobic compounds would have much utility. The decreased toxicity and potentially enhanced bioavailability of PEG-based solid dispersion formulations could mitigate some of the challenges associated with compound administration for a wide range of studies involving *D. melanogaster* as a model system. The cost effectiveness and straight forward synthesis of the formulation compared to more complex nanoparticle formulations makes solid dispersions a convenient and cost effective method for administrating widely used hydrophobic compounds, such as RU486 to *D. melanogaster.*

## Supplementary Material

Supplemental Material

## Data Availability

Data will be made available upon request.
