# Supplementary material for: Enhancing drug administration in Drosophila melanogaster: a method for using solid dispersions for improved solubility and bioavailability
Source: Fly (Austin). 2025 Apr 25;19(1):2497565. doi: 10.1080/19336934.2025.2497565 (PMC12036485; doi:10.1080/19336934.2025.2497565)
Supplement: Supplemental Material [file KFLY_A_2497565_SM6076.docx]

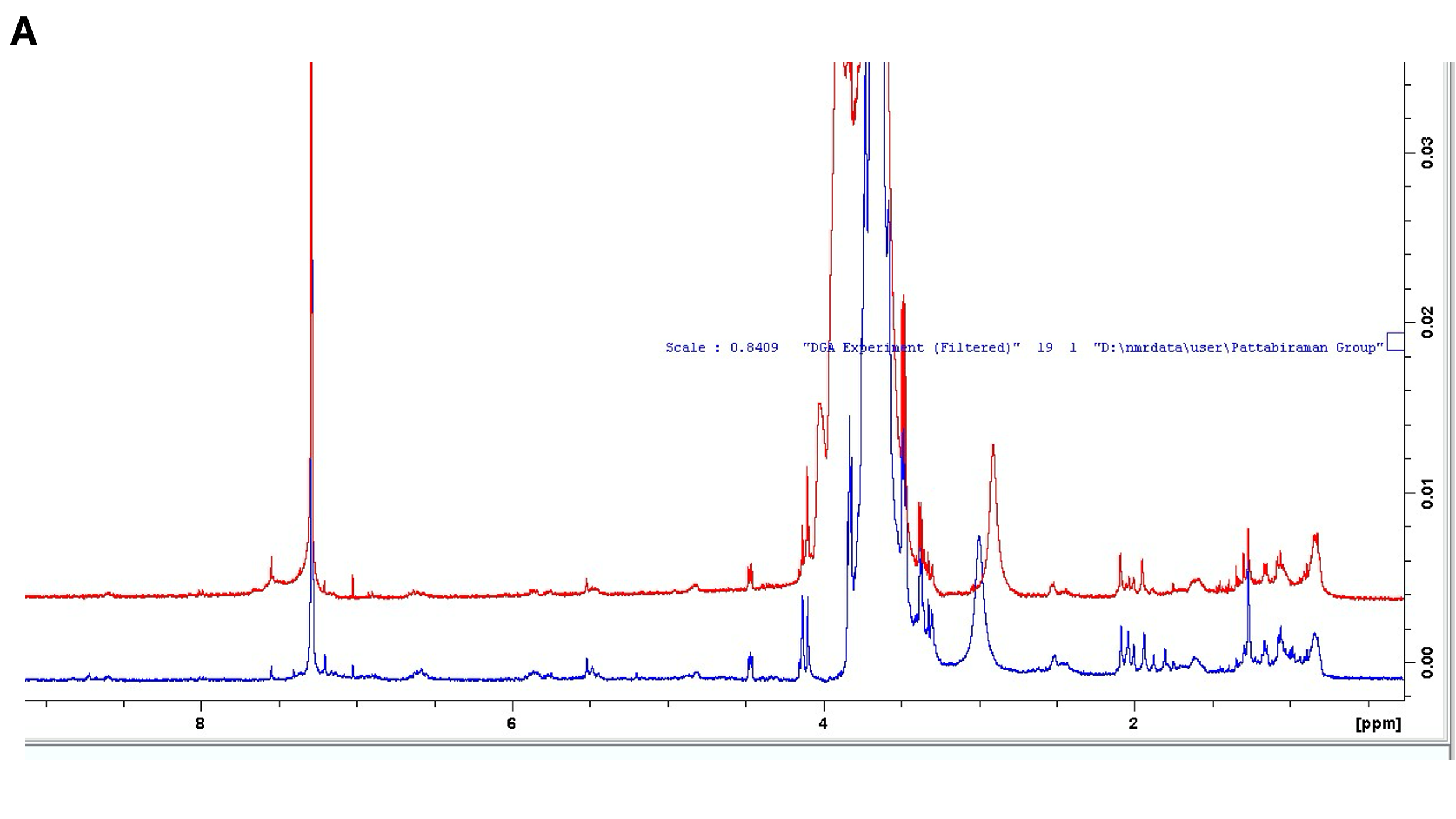


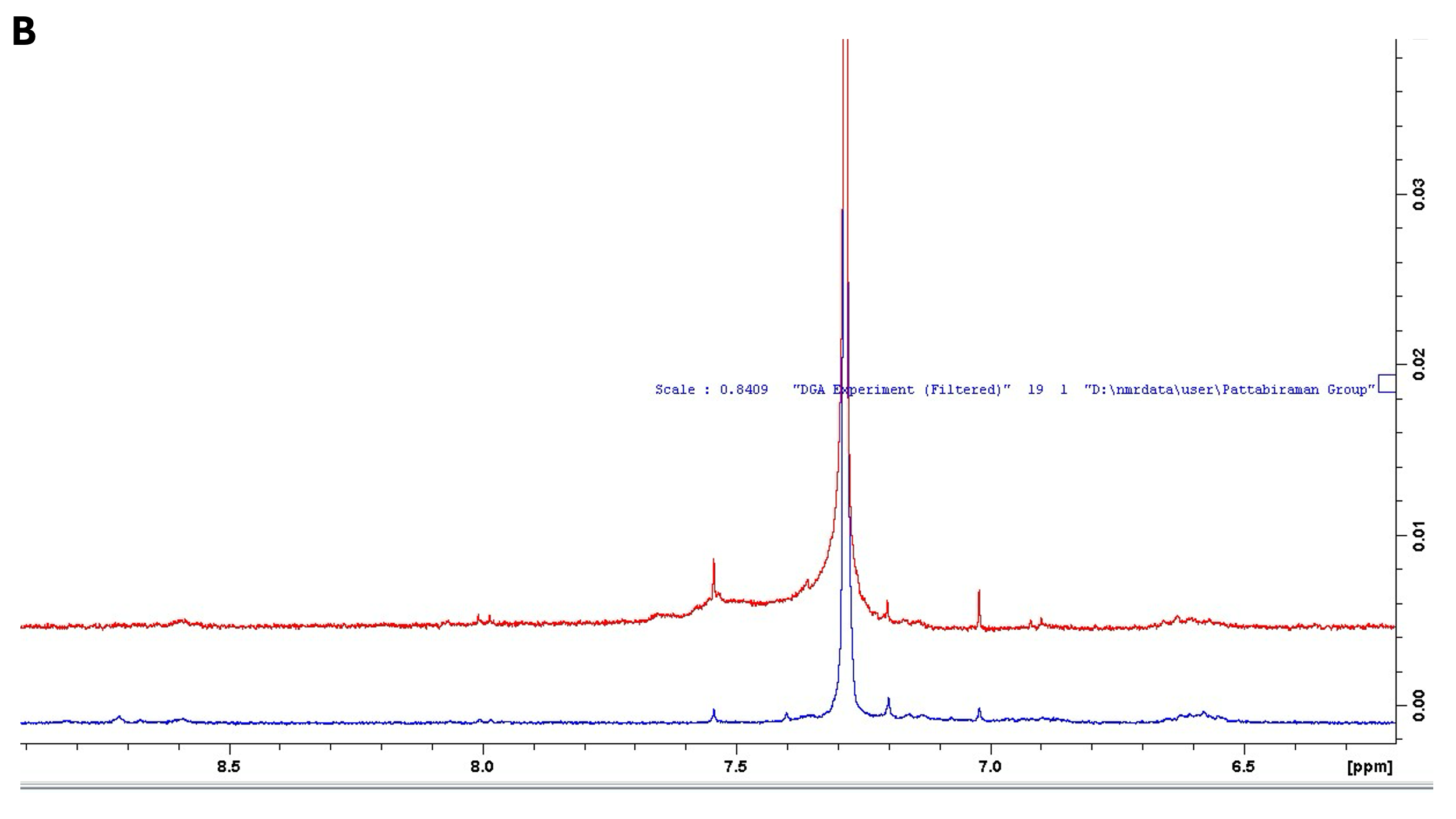


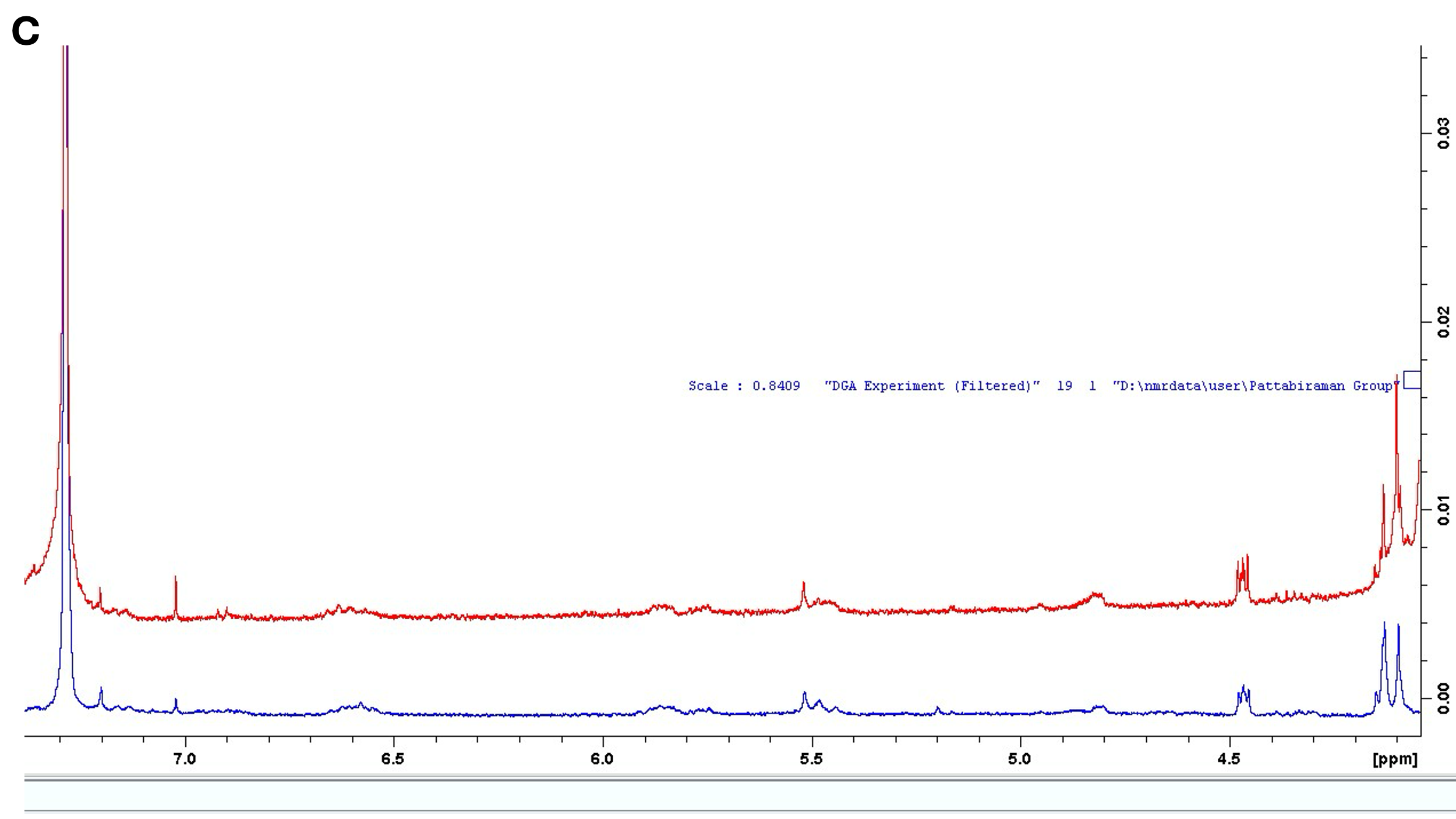


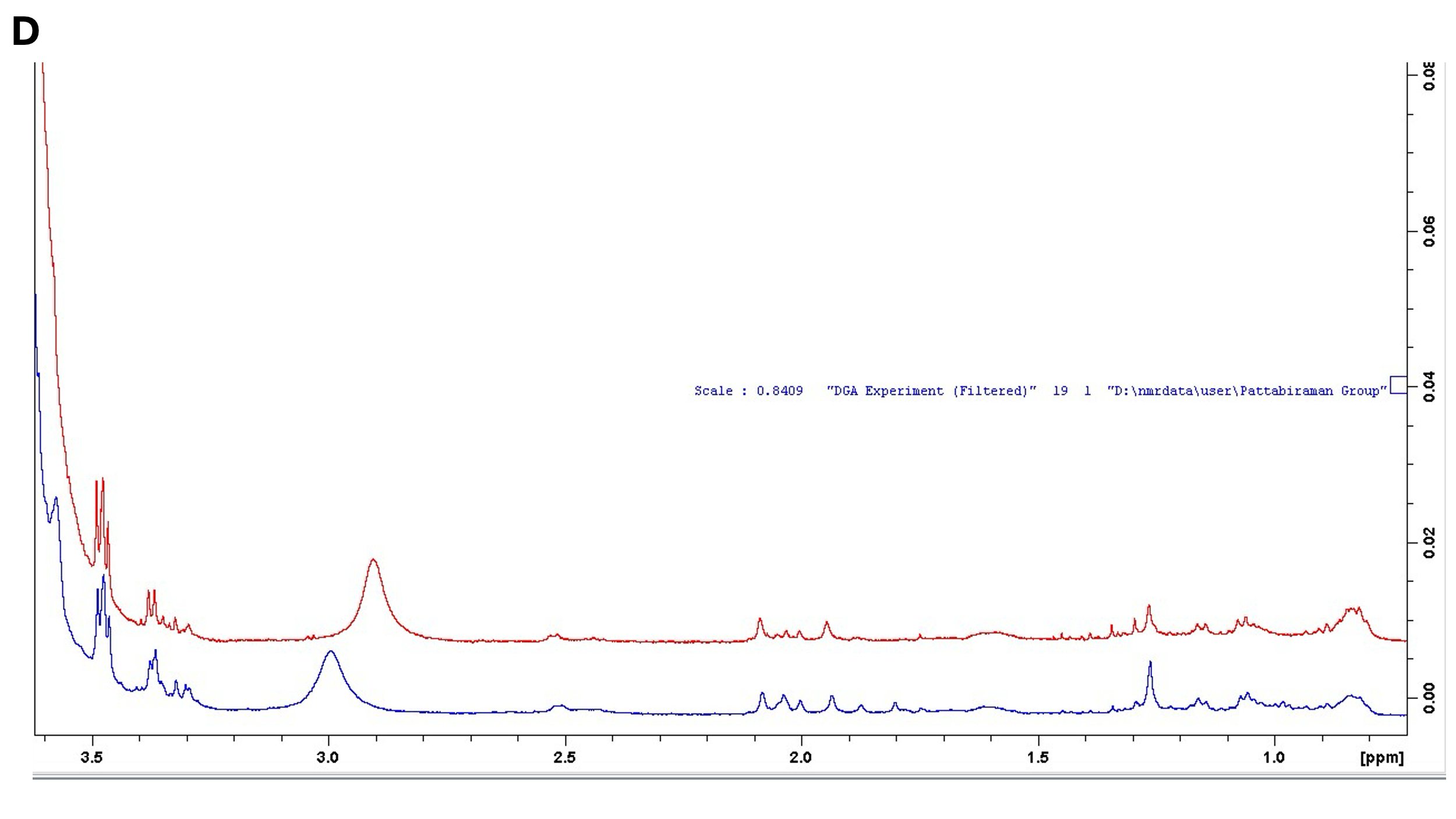


**Supplemental Fig 1**- ^1^H NMR spectra of the accelerated stability test for GA-PEG solid dispersion in CDCl3. Red represents sample that was heated for 1hr at 55°C whereas blue represents freshly prepared sample. The peaks on the spectra remain unchanged throughout indicating the formulation is stable to heating at 55°C.


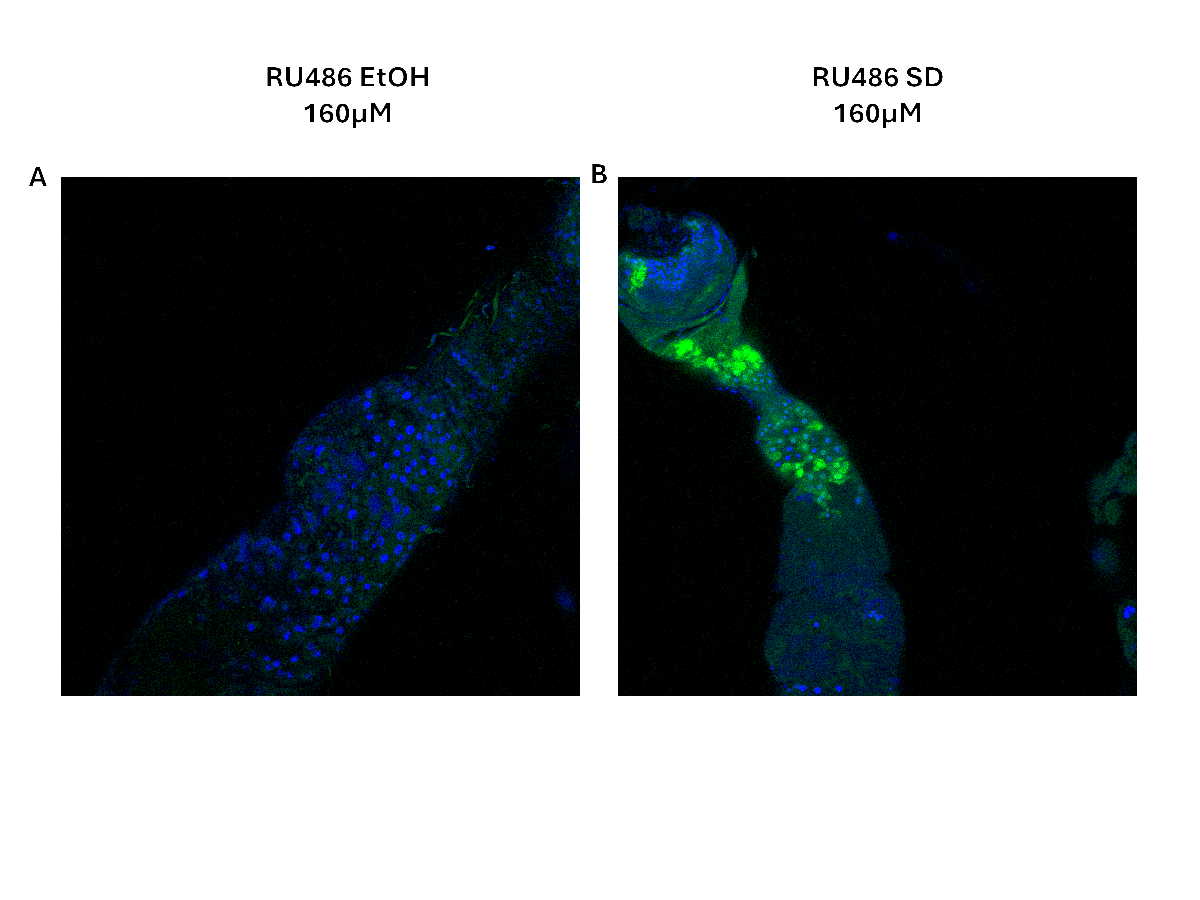


**Supplemental Fig 2-** All images were taken using a 20x objective. (A) fly gut section treated with 160 µM RU486 dissolved in EtOH, and (B) fly gut section treated with 160 µM RU486 in solid dispersion formulation. DAPI staining for nuclei (blue), GFP (green).
